# Effect of the Electrolyte on the Oxygen Reduction Reaction with a MOF Embedded Co‐Porphyrin

**DOI:** 10.1002/cssc.202402295

**Published:** 2025-01-30

**Authors:** Dana Rademaker, Stefania Tanase, Dennis G. H. Hetterscheid

**Affiliations:** ^1^ Leiden Institute of Chemistry Leiden University 2300 RA Leiden The Netherlands; ^2^ Van't Hoff Institute for Molecular Sciences Universiteit van Amsterdam 1098 XH Amsterdam The Netherlands

**Keywords:** metal-organic framework, electrocatalysis, oxygen reduction, cobalt porphyrin, PCN-224

## Abstract

Electrocatalysis in metal‐organic frameworks is an interplay between the diffusion of charges, the intrinsic catalytic rate, and the mass‐transport of reactants through the pores. Here a systematic study is carried out to investigate the role of the electrolyte nature and concentration on the oxygen reduction reaction (ORR) with the PCN‐224(Co) MOF in aqueous electrolyte. It was found that the ORR activity is slightly influenced by the nature of the ions in solution, providing that the ionic strength is high enough to minimize the resistivity during the measurement. The ORR activity was found to be 1.3–1.5 times lower in lithium acetate compared to sodium acetate, while the ORR activity in cesium acetate was 1.3–1.6 times higher compared to the activity in sodium acetate. Moreover, there was no dependency found of the ORR catalysis on the size of the anion, buffer concentration, or oxygen concentration. These findings suggest that ORR catalysis in PCN‐224(Co) is limited by the intrinsic ORR rate at the active site rather than charge transport through the porous structure or substrate transport in the pores. Therefore, optimization of ORR catalysis with this MOF might be achieved by the optimization of the electronics at the cobalt active site.

## Graphical abstract

The reduction of dioxygen to hydrogen peroxide in the PCN‐224(Co) MOF is limited by the intrinsic catalytic activity of the cobalt sites rather than the diffusion of charge or the mass‐transport of reagents. The more strongly coordinating cations affect the catalytic activity slightly negatively, while the concentrations of buffer and oxygen concentration and the radii of the ions in the electrolyte do not affect catalytic activity significantly.

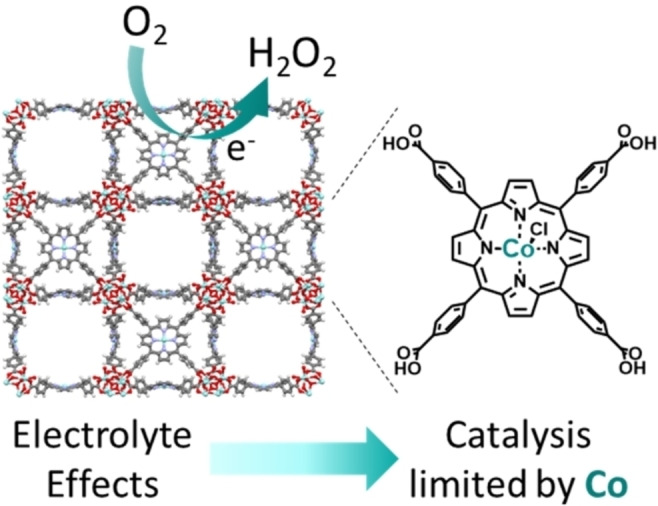

## Introduction

**Figure 1:**
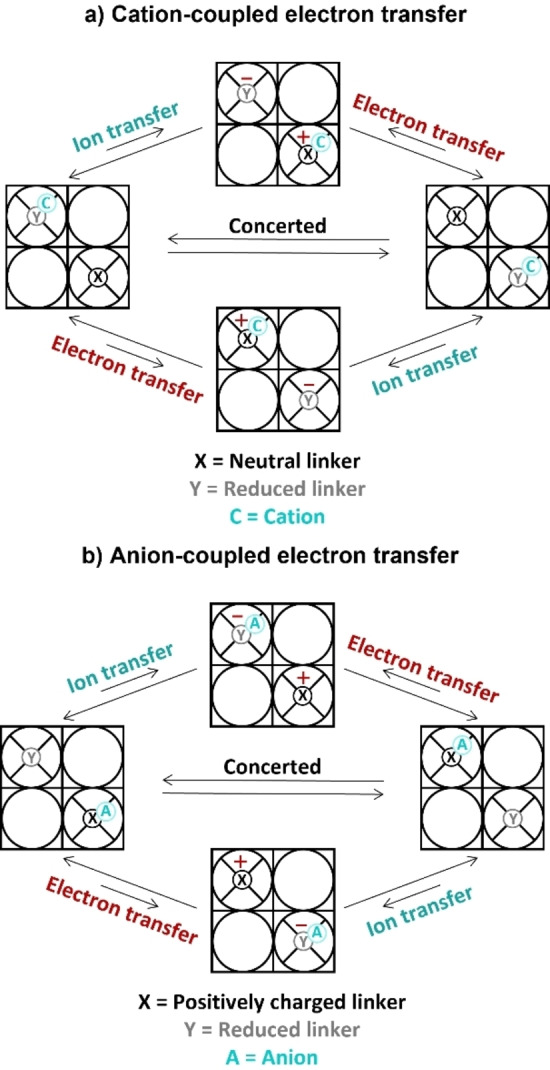
(a) Schematic overview of cation‐coupled electron transfer in a MOF with neutral linkers. The electron transfer from one linker to the next is accompanied by the transfer of a cation to maintain neutrality via a stepwise or concerted mechanism. (b) Schematic overview of anion‐coupled electron transfer in a MOF with cationic linkers. The electron transfer from one linker to the next is coupled to the transfer of an anion in opposite direction to maintain neutrality via a stepwise or concerted mechanism.

Metal‐organic frameworks (MOFs) are three dimensional structures composed of organic linkers and inorganic nodes that are becoming popular scaffolds for the heterogenization and compartmentalization of catalytic sites in redox applications.[^1^, ^2^, ^3^, ^4^, ^5^, ^6^] In the past decade, a substantial amount of work has focused on preparing electroactive MOFs that can conduct electrons for electrochemical applications.[^7^, ^8^] For effective reductive electrocatalysis, electrons must be conducted through the framework toward the active site. This long‐range electron transport between the catalytic sites can be induced by incorporation of redox‐active moieties as node, linker, or guest.[^9^, ^10^] For several MOFs containing electroactive linkers it was proposed that the mode of electron conduction in such MOFs is charge propagation via redox‐hopping between the electroactive sites (Figure 1).[^11^, ^12^, ^13^, ^14^, ^15^, ^16^, ^17^, ^18^] For such MOFs it was found that the redox couple of the electroactive moiety was diffusion limited. This diffusion limited behavior of a heterogeneous catalyst was explained by the coupling of electron‐hopping to the migration of counter ions to balance the charge in organic media.[^7^, ^19^, ^20^] To achieve the charge transfer of the electron in such a MOF, the counter‐ions must reach the electroactive moiety within the porous structure. Since this mass transfer of ions can be slow, movement of the counterion might be the limiting step of the overall charge transport in MOFs.[^7^, ^19^]

Several studies into the effect of counterions and electrolyte on charge transfer rates have been carried out on mainly thin films of MOFs on fluorine‐doped tin‐oxide (FTO) electrodes in organic solvents. Ott *et al*. investigated the diffusion coefficient of cations when using the Zr(dcphOH‐NDI) MOF with redox‐active naphthalenediimide (NDI) linkers.^[12]^ They investigated the mode of transport of a cation upon reduction of the NDI linker in dimethylformamide solution. The apparent diffusion coefficient was shown to decrease by one order of magnitude upon changing from LiClO_4_ as electrolyte salt to TBAPF_6_ (TBA^+^=tetrabutylammonium, PF_6_^−^=hexafluorophosphate). The effect of different pore sizes on the charge propagation was investigated by Morris *et al*. for ferrocene‐doped MOFs with pore sizes of 15 Å (MOF‐808), 33 Å (NU‐1000), and 47 Å (NU‐1003).^[21]^ Electron and ion diffusion rates were determined in acetonitrile electrolyte with either TBAPF_6_ or TBATFAB (TFAB^−^=tetrakis(pentafluorophenyl) borate) as the electrolyte. In general, it was found that with increasing pore size the ion diffusion coefficient increased and the electron diffusion coefficient decreased. Overall, the charge transfer rate increased when the pore size increased. Moreover, comparison of the different anions indicated that when the smaller PF_6_^−^ (3.6 Å) was used instead of the large TBAF^−^ (10.3 Å), the ion diffusion coefficient is found to be higher for MOF‐808 and NU‐1000. However, when the larger NU‐1003 was used, the rate of ion diffusion became independent of the size of the counterion. The results indicated that the charge transfer rate does not depend on the size of the counter ion when the pore size is at least four times larger than the ion size. These studies indicate that in organic solvents the charge transfer rate is governed by the counter‐ion diffusion and the counter‐ion size relative to the pore size. However, when an electroactive MOF should function as a catalyst for small molecule activation in industry, such as the electrochemical oxygen reduction reaction (ORR), the solvent is often water instead of the organic solvents used in the fundamental studies. The polar water molecules can also stabilize charges within the pores, which might decrease the necessity of anion‐ or cation‐coupled electron transfer.^[22]^ Therefore, the dependence of charge transfer on the diffusion of ions is expected to be less in aqueous solution than in organic media. Thus far, electron transfer in MOFs in aqueous electrolyte has not been investigated.

Moreover, two additional parameters besides charge transfer are expected to influence electrocatalysis in MOFs, namely the diffusion of reactants to the active site and the intrinsic catalytic reaction rate at the active site.[^20^, ^23^, ^24^] Depending on what the rate‐limiting factor is for electrocatalysis in MOFs, three scenarios are envisioned:^[20]^

- Diffusion of the substrate(s) is limiting catalysis in the MOF. In the oxygen reduction reaction this could hold for both diffusion of dioxygen and diffusion of protons. When diffusion of either of these reactants is rate limiting, the charge transfer within the MOF and the catalytic rate at the active site are faster than diffusion of the substrate which will lead to a boundary thickness of the MOF that is active for the catalysis that depends on the substrate concentration in the pores. Only the outside surface of the MOF particle will be active, and the substrate is consumed before it can reach the bulk of the particle. If this scenario occurs, there is no dependence of catalysis on the nature or concentration of the ions in solution expected.
- The intrinsic catalytic rate is limiting catalysis in the MOF. When this is the case the charge transfer within the MOF and the diffusion of reactants are faster than the consumption of the reactants at the active site. In such a scenario, catalysis is expected to occur throughout the entire MOF and is only dependent on the rate‐determining step of the catalytic mechanism. If this scenario occurs, there should be no dependence of catalysis on the nature or concentration of the ions in solution unless they are involved in the rate‐determining step of catalysis.
- Charge transfer between the electroactive sites is limiting catalysis in the MOF. When this is the case, the diffusion of substrate and the catalytic rate at the active site are faster than the charge transfer towards the active site. If this occurs, the catalysis will mainly occur at the boundaries between the electrode material and the electrolyte. In this scenario, a dependence of catalysis on the concentration and nature of the ions that accompany electron transfer is expected.

We recently reported PCN‐224(Co) as an active, selective, and stable catalyst for the electrochemical ORR in an aqueous sodium acetate buffer solution (pH 4.7).^[25]^ To find out which scenarios are important for electrocatalysis in MOFs in aqueous solution herein a systematic study is carried out that sets out to investigate the role of the electrolyte nature and concentration on the electrocatalytic ORR with the PCN‐224(Co) MOF as catalyst.

## Results

### Synthesis of PCN‐224(Co)

**Figure 2:**
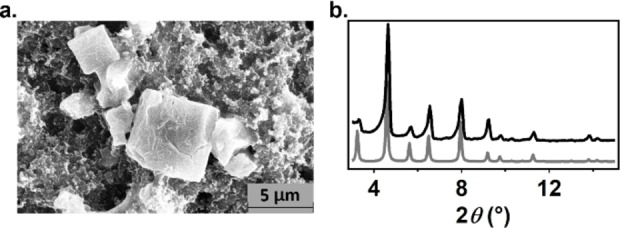
(a) SEM image of PCN‐224(Co) ink measured at 15 kV and 0.1 nA. (b) PXRD pattern PCN‐224(Co) synthesized in this work (black) and the theoretical spectrum of PCN‐224 (grey).

PCN‐224(Co) consists of Zr_6_(O)_4_(OH)_4_(H_2_O)_6_ nodes connected by cobalt 5,10,15,20‐(4‐carboxyphenyl)‐porphyrin chloride (CoTCPP) linkers. The framework PCN‐224(Co) was produced via a solvothermal synthesis by combining ZrCl_4_ and CoTCPP in DMF with benzoic acid as modulator as reported previously.^[25]^ The framework that was formed was characterized with scanning electron microscopy (SEM), powder X‐ray diffraction (PXRD), and inductively coupled plasma mass spectrometry (ICP‐MS). The SEM images indicate a cubic morphology of the particles in the range of 1–5 μm. This cubic structure is in agreement with the PCN‐224 morphology (Figure 2a).^[26]^ The PXRD pattern shows the reflections of phase‐pure PCN‐224 (Figure 2b).[^26^, ^27^] Moreover, ICP‐MS analysis indicates 1.55±0.04 Co ions per Zr_6_ node, which agrees with the Zr_6_(O)_4_(OH)_4_(H_2_O)_6_(CoTCPP)_1.5_ unit cell of PCN‐224(Co). Lastly analysis of the N_2_‐isotherm indicated pore sizes of 8 and 20 Å, indicative of PCN‐224. Combined these analytical data for PCN‐224(Co) are in good agreement with a PCN‐224(Co) sample of high crystallinity.

### The Effect of Buffer Concentration and Ionic Strength on the ORR with PCN‐224(Co)

**Figure 3:**
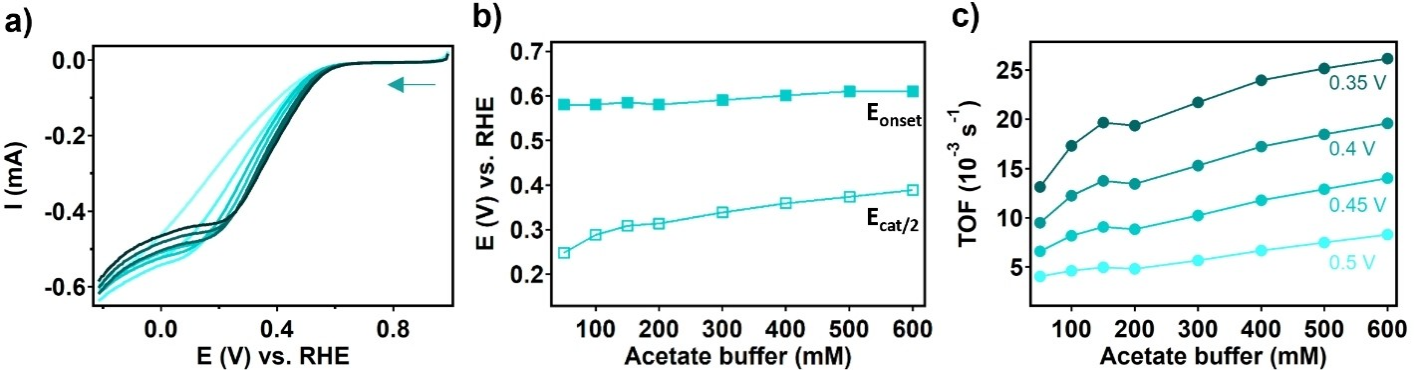
(a) RDE LSV traces of PCN‐224(Co) in a sodium acetate buffer (pH 4.7) with concentrations of (from light to dark) 50, 100, 150, 200, 300, 400, 500, and 600 mM. Analysis of RDE LSV traces of PCN‐224(Co) with (b) E_onset_ and E_cat/2_ as a function of the sodium acetate buffer concentration and (c) the TOF at 0.5, 0.45, 0.4, and 0.35 V vs. RHE as a function of the sodium acetate buffer concentration. Measured with 50 mV/s scan rate and 1600 rpm rotation rate under an oxygen atmosphere.

**Figure 4:**
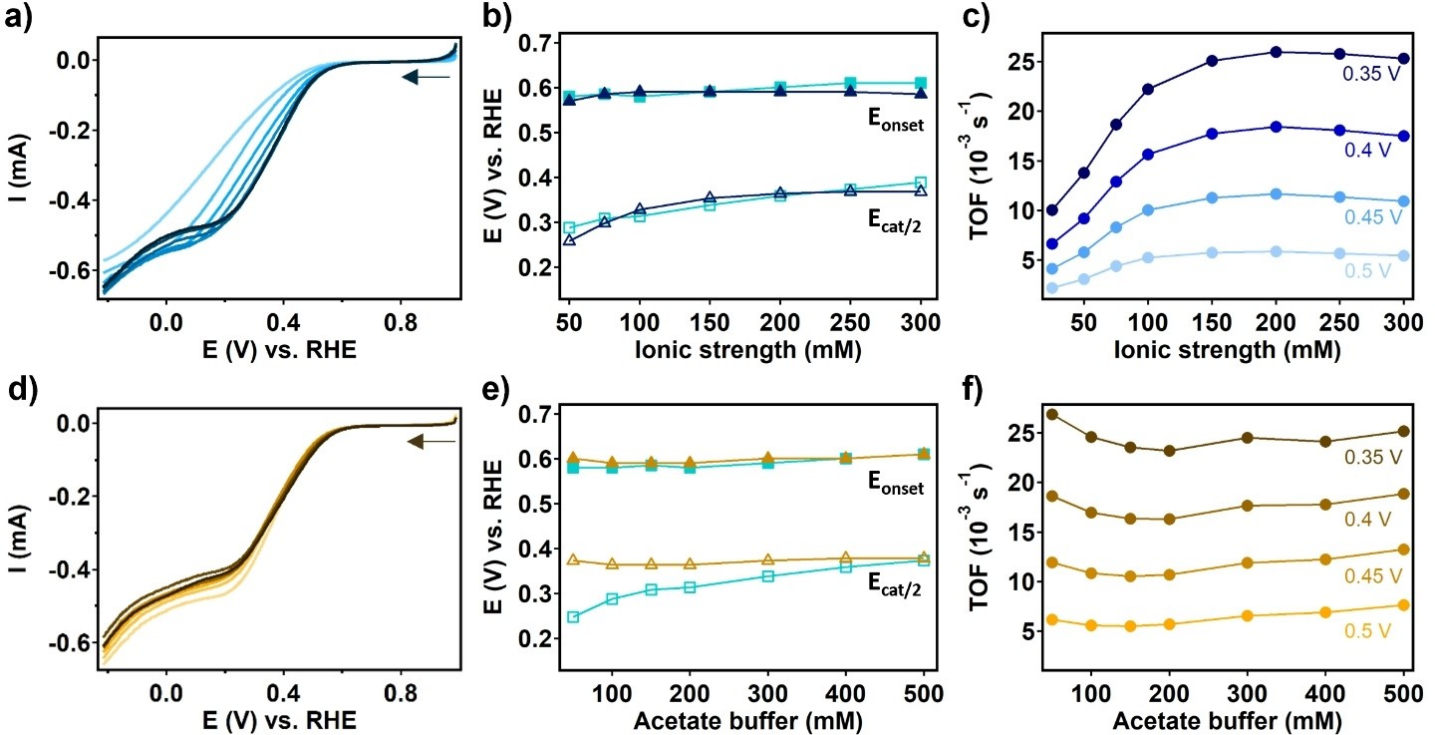
(a) RDE LSV traces of PCN‐224(Co) in 50 mM sodium acetate buffer (pH 4.7) with increasing NaNO_3_ concentrations of 0, 25, 50, 75, 125, 175, 225, and 275 mM (from light to dark). Analysis of RDE LSV traces of PCN‐224(Co) with (b) E_onset_ and E_cat/2_ as a function of the ionic strength of the sodium acetate dependence between 50–300 mM ionic strength of sodium acetate buffer (teal) and the ionic strength dependence with 50 mM of sodium acetate and NaNO_3_ as supporting electrolyte (dark blue). (c) The TOF at 0.35, 0.4, 0.45, and 0.5 V vs. RHE as a function of the ionic strength with a constant 50 mM of sodium acetate and NaNO_3_ as supporting electrolyte. (d) RDE LSV traces of PCN‐224(Co) in an electrolyte of NaNO_3_ and sodium acetate buffer at a constant ionic strength of 300 mM, while changing the sodium acetate buffer to 50, 100, 150, 200, 300, 400, and 500 mM (from light to dark). Analysis of RDE LSV traces of PCN‐224(Co) with (e) E_onset_ and E_cat/2_ as a function of the sodium acetate buffer concentration between 50–300 mM ionic strength of sodium acetate buffer (teal) and the sodium acetate buffer dependence with supporting NaNO_3_ electrolyte to achieve a fixed ionic strength of 300 mM (orange). (f) The TOF at 0.35, 0.4, 0.45, and 0.5 V vs. RHE as a function of the sodium acetate buffer concentration of the sodium acetate buffer dependence with supporting NaNO_3_ electrolyte to achieve a fixed ionic strength of 300 mM. LSV measurements were carried out with 50 mV/s scan rate and 1600 rpm rotation rate under an oxygen atmosphere.

**Figure 5:**
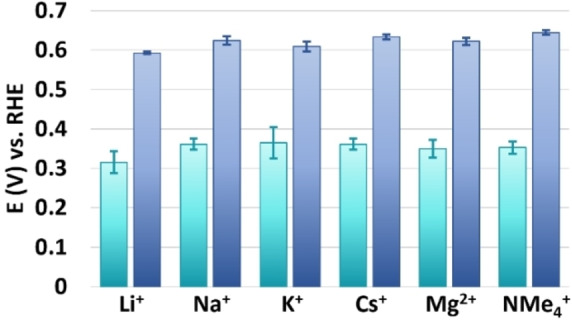
E_onset_ (blue) and E_cat/2_ (teal) analysis of RDE LSV traces of PCN‐224(Co) in 0.25 M ionic strength acetate buffer with Li^+^, Na^+^, K^+^, Cs^+^, Mg^2+^, and NMe_4_^+^ as cationic species. Error bars indicate the standard deviation of four measurements.

To heterogenize the MOF on the working electrode, an ink was made of the PCN‐224(Co) catalyst suspended with carbon black (CB) as an electron conducting additive and Nafion as an adhesive binder to maintain the physical intactness of the dropcast. This ink was dropcasted onto a glassy carbon electrode and allowed to dry in air. The effect of electrolyte concentration on the ORR activity with PCN‐224(Co) was assessed with rotating disk electrode linear sweep voltammetry (RDE LSV) in an aqueous sodium acetate buffer solution (pH 4.7) under an oxygen atmosphere (Figure 3a). LSV measurements were conducted while the concentration of the sodium acetate buffer was increased from 50 to 600 mM. The onset potential (E_onset_), the catalytic half‐wave potential (E_cat/2_) and the turn‐over frequency (TOF) were selected as the main descriptors to assess the changes in the LSV curves as a function of the acetate buffer concentration (Figure 3b and 3c). The difference between E_onset_ and E_cat/2_ indicates how steep the increase of the catalytic current is. The TOF indicates the number of product molecules generated per cobalt porphyrin site per second (SI 2). For the determination of the TOF we assumed that all cobalt centers present in the MOF contribute equally, yet that is not necessarily the case as entire MOF particles may be deeply buried underneath a carbon black layer. The TOFs at 0.35, 0.4, 0.45, and 0.5 V vs. RHE are determined as a function of the acetate buffer concentration and plotted in Figure 3c. For PCN‐224(Co), E_cat/2_ increases with increasing sodium acetate buffer concentration from 0.25 to 0.39 V vs. RHE between 50 and 600 mM sodium acetate buffer, while E_onset_ only changes slightly from 0.58 to 0.61 V vs. RHE upon this increase of the electrolyte concentration. Moreover, the TOF doubles when increasing the sodium acetate buffer concentration from 50 to 600 mM. Both phenomena indicate that the ORR rates increase with the buffer concentration. This observation can be caused by two different variables that are changed when the sodium acetate buffer concentration is increased, i. e. either the increase of the concentration of the buffering species or the increase of the ionic strength of the solution. To verify which electrolyte phenomenon is responsible for the changes seen when the sodium acetate buffer concentration is increased, RDE LSV measurements were carried out with PCN‐224(Co) in a 50 mM sodium acetate buffer solution where NaNO_3_ was added to increase the ionic strength from 25 to 300 mM (Figure 4a), and in a solution where the sodium acetate buffer concentration is increased between 50 and 500 mM while a constant ionic strength of 300 mM is maintained by supplying NaNO_3_ (Figure 5d). From the LSV traces, the E_onset_, E_cat/2_, and TOF were determined and compared to the data obtained for the sodium acetate buffer dependence study without added NaNO. E_cat/2_ increases from 0.23 to 0.37 V vs. RHE upon increasing the ionic strength from 25 to 300 mM, and the TOF increases 2.5 times upon this increase in ionic strength (Figure 5b and 5c). On the other hand, when the ionic strength is kept constant while the sodium acetate buffer component of the electrolyte is increased, E_onset_, E_cat/2_, and the TOF appear unaffected (Figure 5e and 5f). These observations point to the ionic strength as the main effector for the increased catalytic ORR rather than the buffering strength of the solution.

To find out if the necessity for a high ionic strength for ORR catalysis with PCN‐224(Co) is due to the porous structure of the MOF, or whether this is inherent to ORR catalysis with cobalt porphyrins, the buffer and electrolyte dependency studies were also carried out for CoTCPP dropcasted on the electrode (SI 3). RDE LSV measurements were carried out in a 50 to 600 mM sodium acetate buffer solution, in a 50 mM sodium acetate buffer solution where NaNO_3_ was added to increase the ionic strength from 25 to 300 mM, and in a solution where the sodium acetate buffer concentration is increased between 50 and 500 mM while a constant ionic strength of 300 mM is maintained by supplying NaNO_3_ (Figure S1). From these LSV traces the E_onset_ and E_cat/2_ were calculated (Figure S2), as well as the TOFs at 0.35, 0.4, 0.45, and 0.5 V vs. RHE (Figure S3). Upon increasing the sodium acetate buffer concentration from 50 to 600 mM, E_cat/2_ increases from 0.43 to 0.57 V vs. RHE, while E_onset_ remains stable at 0.69 V vs. RHE. When the sodium acetate buffer concentration was kept constant at 50 mM and the ionic strength was increased with NaNO_3_ a similar trend was observed of increasing E_cat/2_ with increasing ionic strength. When the ionic strength was kept constant at 300 mM while increasing the sodium acetate buffer concentration, E_cat/2_ remained 0.52 V vs. RHE and E_onset_ remained 0.69 V vs. RHE while increasing the sodium acetate buffer concentration from 50 to 500 mM. Furthermore, the TOF doubled when increasing the sodium acetate buffer concentration from 50 to 600 mM or when the sodium acetate buffer concentration was kept constant at 50 mM and the ionic strength was increased with NaNO_3_. When the ionic strength was kept constant and the sodium acetate buffer concentration was increased, the TOF showed to be independent of the acetate buffer concentration. These findings for ORR with CoTCPP suggest that there is a dependence of the catalysis on the ionic strength for the CoTCPP porphyrin. Therefore, this dependence of the ORR on the ionic strength must be due to a lowering of the resistivity in the electrochemical system and is probably not related to the porous structure of the MOF.

### Effect of the Ion Identity on ORR with PCN‐224(Co)

**Figure 6:**
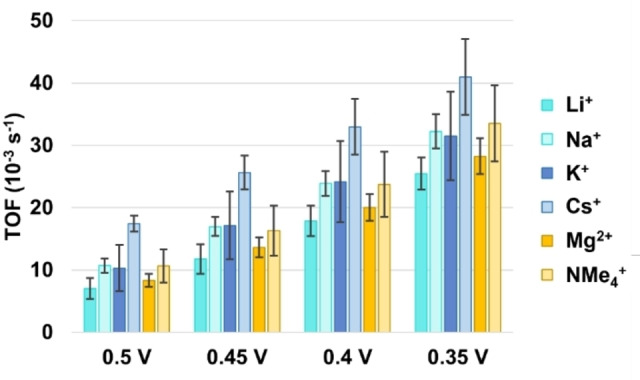
TOF analysis of RDE LSV traces of PCN‐224(Co) in 0.25 M ionic strength acetate buffer with Li^+^, Na^+^, K^+^, Cs^+^, Mg^2+^, and NMe_4_^+^ as cationic species at 0.5; 0.45; 0.4; and 0.35 V vs. RHE. Error bars indicate the standard deviation of four measurements.

To investigate the role of cation transport during ORR catalysis with PCN‐224(Co), RDE LSV studies were carried out in a 0.25 M ionic strength acetate buffer solution with lithium, sodium, potassium, cesium, magnesium, or tetramethylammonium as cationic species. For each condition, RDE LSV measurements were performed for four dropcasts to account for variability (Figure S4). From these LSV traces, the E_onset_ and E_cat/2_ were calculated (Figure 5) as well as the TOFs at 0.5, 0.45, 0.4, and 0.35 V vs. RHE (Figure 6 and Table S1). The ORR with PCN‐224(Co) in sodium acetate results in a E_onset_ of 0.62±0.1 V vs. RHE and E_cat/2_ of 0.36±0.01 V vs. RHE. The values of E_onset_ and E_cat/2_ based on ORR with PCN‐224(Co) in potassium, cesium, magnesium, and tetramethylammonium acetate are within the statistical error of the measurements in sodium acetate. Only the ORR measurements in lithium acetate led to a lower E_onset_ of 0.59±0.003 and E_cat/2_ of 0.32±0.03 V vs. RHE. Moreover, the TOF increases with increasing overpotential in all acetate buffer solutions. The TOF values for ORR in sodium acetate at all potentials is within statistical error of the values found in potassium, magnesium, and tetramethylammonium acetate. The TOF in lithium acetate is lower at each potential compared to the ORR in sodium acetate, while the TOF in cesium acetate is higher between 0.5–0.4 V vs. RHE.

**Figure 7:**
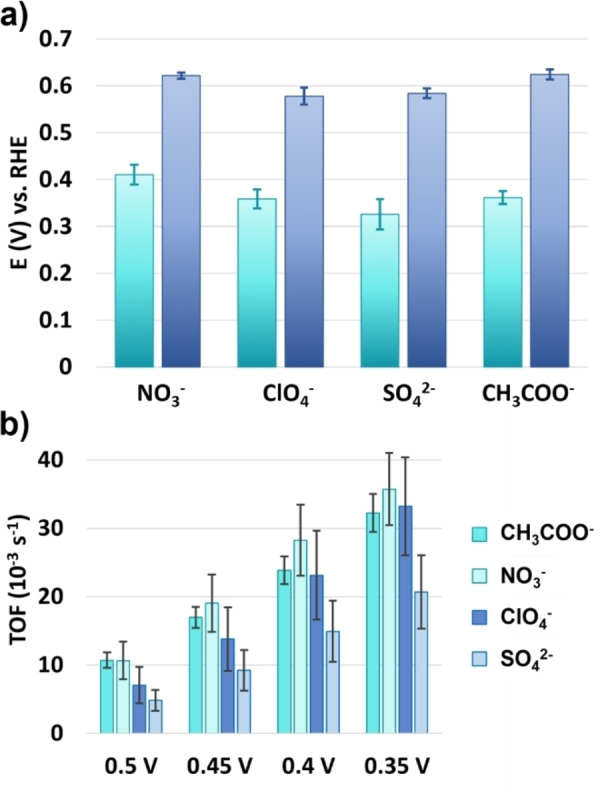
(a) E_onset_ (blue) and E_cat/2_ (teal) analysis of RDE LSV traces of PCN‐224(Co) in 50 mM acetate buffer with different anionic electrolyte additives. (b) TOF analysis of RDE LSV traces of PCN‐224(Co) in 50 mM acetate buffer with different anionic electrolyte additives at 0.5; 0.45; 0.4; and 0.35 V vs. RHE. Error bars indicate the standard deviation of four measurements.

To investigate the role of anion transport during ORR catalysis with PCN‐224(Co), RDE LSV studies were carried out in solutions with 50 mM sodium acetate buffer while adding sodium nitrate, sodium perchlorate, or sodium sulfate electrolyte to reach a 250 mM ionic strength solution (Figure S5). The sodium acetate buffer was needed for stability of the framework during ORR catalysis. Without the buffering species present, the MOF decomposes and leaches from the electrode during the LSV measurement. From the LSV traces, the average E_onset_, E_cat/2_ and TOFs at 0.5, 0.45, 0.4, and 0.35 V vs. RHE were calculated (Figure 7 and Table S2). The E_cat/2_ for measurements in acetate, nitrate, perchlorate, and sulfate containing solutions was found to be 0.36, 0.41, 0.36, and 0.33 V vs. RHE, respectively. Of these values, only the E_cat/2_ of the measurement with nitrate shows a significant increase compared to the ORR in acetate electrolyte. This increase is reflected in the higher TOF values for ORR in nitrate solution at the potentials between 0.45–0.35 V vs. RHE as well, although this increase falls within the statistical error of the TOFs measured in acetate solution. For the TOF values only a decrease is seen when sulfate is used as the ionic species in solution during the ORR.

## Discussion

**Figure 8:**
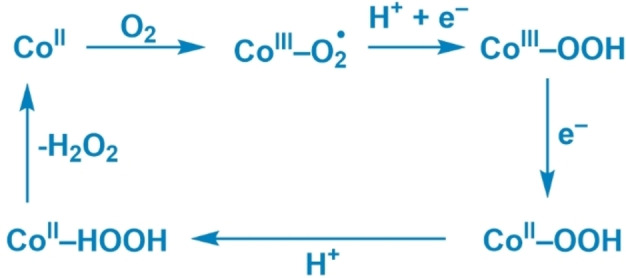
General ORR mechanism of cobalt porphyrin‐based catalysts.

**Figure 9:**
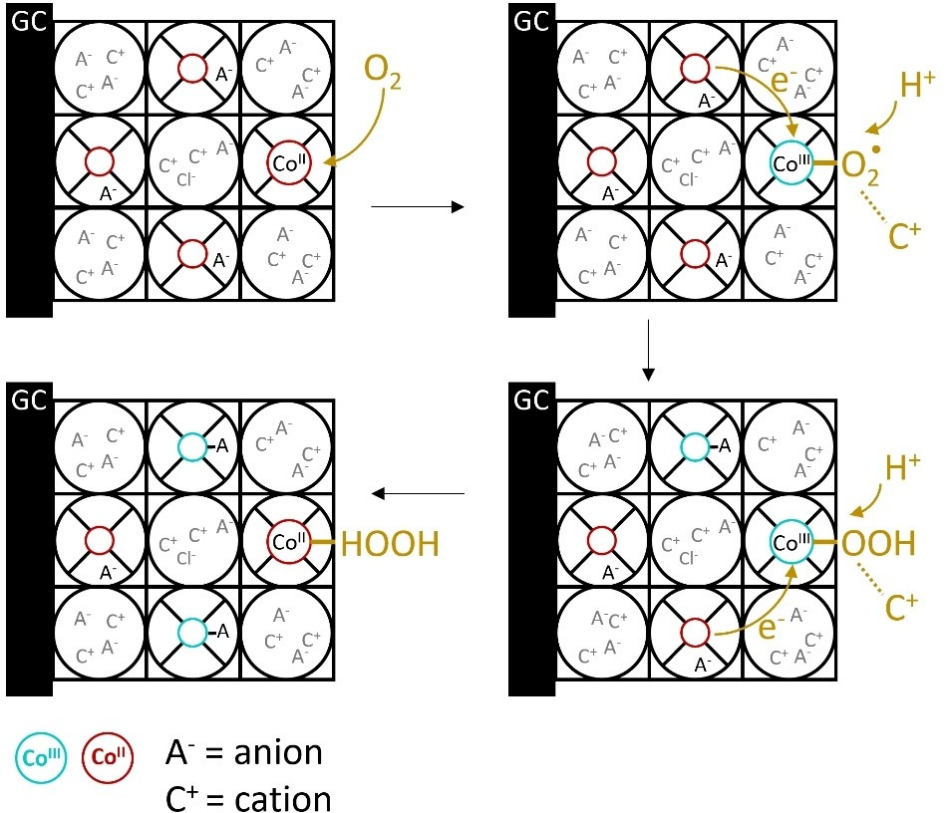
Schematic overview of the expected charge transfer mechanism in PCN‐224(Co). Electron (e^−^) transfer occurs between the electrode and the linker of the MOF, and between the linkers. Upon reduction of a cobalt site to Co^II^, the coordinated anion dissociates and diffuses in the pores. At the same time, the site that supplied the electron is oxidized to a Co^III^ species and an anion is coordinated.

During the ORR in PCN‐224(Co) catalysis can be limited by the diffusion of charges, the mass transfer of dioxygen or protons, or the intrinsic catalytic rate of the ORR at the cobalt sites. From the ORR mechanism at the cobalt site it is known that the first proton‐coupled electron transfer step is often rate‐determining, while the second electron transfer is generally the potential‐determining step (Figure 8).[^28^, ^29^, ^30^, ^31^, ^32^, ^33^, ^34^, ^35^, ^36^] After the first electron transfer the hydroperoxo species is strongly bound in the Co^III^‐OOH intermediate and the transfer of another electron can be either an uphill process or a downhill process with a low free energy gain. The electron transfer throughout the PCN‐224(Co) framework that likely proceeds via a stepwise manner via individual redox sites must be able to supply the thermodynamic driving force for this second electron transfer. At the start of catalysis, PCN‐224(Co) contains Co^III^‐Cl porphyrin sites. These sites can be reduced to allow charge transfer through the framework by the Co^III^/Co^II^ couple.^[37]^ Upon electron transfer between the cobalt sites, it is expected that anion transfer occurs in the opposite direction to maintain charge neutrality. Maintaining charge neutrality in electron transfer through MOFs appears to be very relevant in organic solvent, while to a lesser extend explored in aqueous solutions. Upon reduction of a cobalt site to Co^II^, the anion is expected to dissociate and diffuse into the pores. At the same time, the site that supplied the electron is oxidized to a Co^III^ species and would have to bind an anion to maintain charge neutrality. The expected charge transfer mechanism in PCN‐224(Co) is shown in Figure 9. From the anion dependence study on ORR with PCN‐224(Co) it was found that the ORR is independent on whether acetate, nitrate, or perchlorate is used in the electrolyte solution, while catalysis is slowed down upon use of sulfate as anion in solution. The TOF values of ORR in acetate solution are 1.6–2.2 times greater than the TOF values found in sulfate solution. This difference cannot be ascribed to the size of the hydrated anion (Table 1), with acetate (2.17 Å) and nitrate (2.23 Å) being significantly smaller than perchlorate (2.69 Å) and sulfate (2.73 Å). The limited differences observed between ORR in acetate and perchlorate solutions indicate that the size of the monovalent anion does not influence the catalysis significantly. Therefore, the charge transfer is either not dependent on the anions or the charge transfer is not the rate‐limiting factor of the catalytic ORR. The lower catalytic rates in sulfate solution might be attributed to the divalent charge of the anion. The divalent anion requires the movement of more cations to allow for electroneutrality within the pores of PCN‐224(Co) than the monovalent anions. Another explanation could be that sulfate might bind the cobalt porphyrin site more strongly and prevents the interaction of the catalyst with oxygen, thereby effectively slowing down catalysis.

**Table 1:** Ionic radius and hydrated radius of aqueous electrolyte ions.[^38^, ^39^]

Ion	Ionic radius (Å)	Hydrated radius (Å)
Li^+^	0.94	3.82
Na^+^	1.17	3.58
K^+^	1.49	3.31
Cs^+^	1.86	3.29
Mg^2+^	0.72	4.28
NMe_4_^+^	2.85	3.47
H_3_O^+^	1.15	2.80
CH_3_COO^−^	1.62	2.17
NO_3_^−^	1.79	2.23
ClO_4_^−^	2.50	2.69
SO_4_^2−^	2.30	2.73

During the ORR in PCN‐224(Co), oxygen mass transfer might also influence the catalytic rate. We previously investigated the ORR with PCN‐224(Co) with an RDE rotation rate dependence in a 0.2 M acetate buffer electrolyte (Figure S6).^[25]^ This rotation rate dependence indicated that the LSV current remained constant in the regime between 0.35–0.5 V vs. RHE, while it increased with increasing rotation rate between −0.2 to 0.2 V vs. RHE. The TOF values in this study are determined in the current regime between 0.5–0.35 V vs. RHE. In this regime close to E_onset_, the mass transfer from the bulk towards the electrode should not be limiting catalysis. The constant LSV current obtained in this regime while increasing the RDE rotation rate suggests that at these potentials another factor is kinetically limiting the catalytic reaction rather than the mass transfer of oxygen towards the electrode. This limiting factor might either be the diffusion of oxygen from the surface of the MOF particle through the pores, or the intrinsic catalytic activity of the active sites. The cation dependence study with several acetate solutions indicated that the TOFs of ORR catalysis with PCN‐224(Co) follows the trend Cs^+^>Na^+^≈K^+^≈NMe_4_^+^≈Mg^2+^>Li^+^_._ It was found that ORR catalysis is 1.3–1.5 times slower in lithium acetate and is 1.3–1.6 times faster in cesium acetate compared to ORR in sodium acetate. These rate differences between ORR catalysis with different cations cannot be correlated with the hydrated ionic radius of the ions (Table 1).

**Figure 10:**
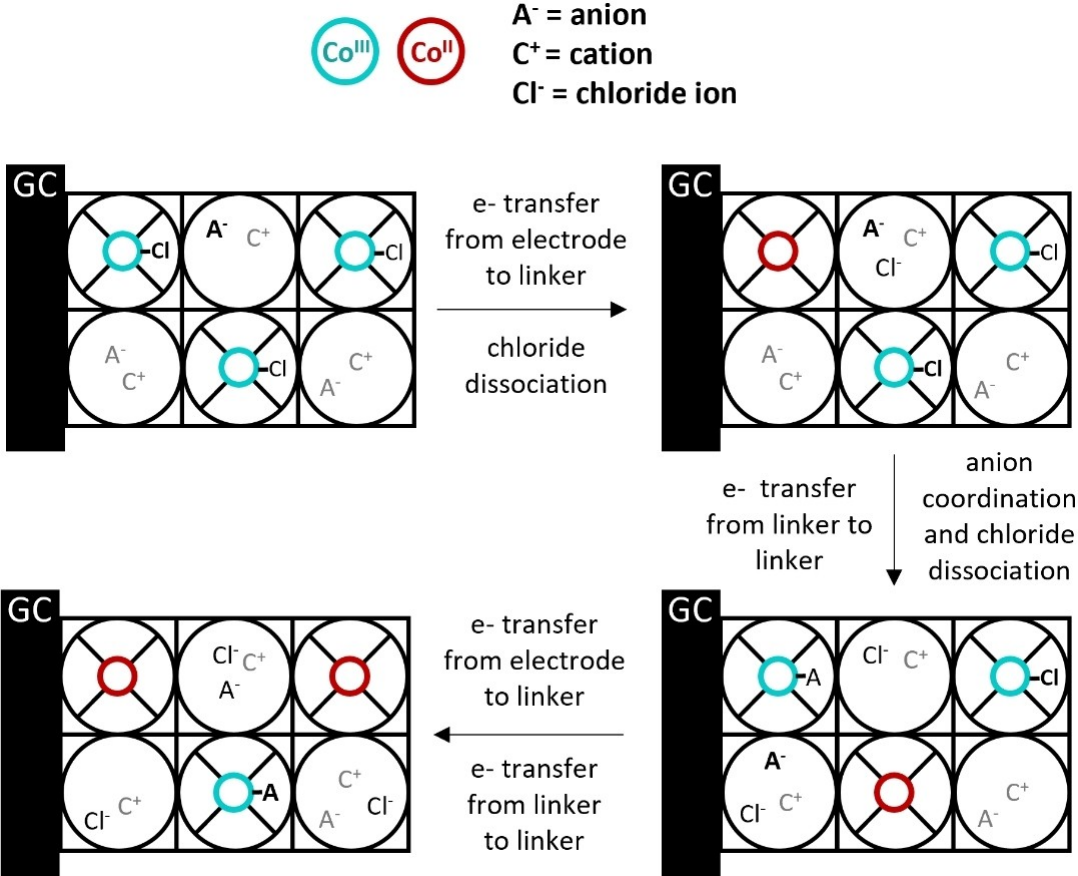
Schematic overview of the expected ORR mechanism in PCN‐224(Co). The charge transfer between the Co sites leads to the formation of Co^II^ sites in the MOF. The Co^II^ site can bind oxygen and form a Co superoxide intermediate. This superoxide intermediate might be stabilized by a cation from the electrolyte. After a proton transfer from within the pores and an electron transfer via electron hopping from another linker toward the active site, a peroxide intermediate is formed. Upon another electron and proton transfer H_2_O_2_ is formed at the active site.

The influence of the cation might be ascribed to a specific interaction of the ion with intermediates during the ORR. In studies by Markovic *et al*. and Nakamura *et al*. cations were found to thermodynamically stabilize hydroxide intermediates on a Pt(111) surface during ORR catalysis in the order Cs^+^<K^+^<Na^+^≈NMe_4_^+^<Li^+^, which leads to the slowest ORR catalysis with lithium as cation in solution and the fastest ORR catalysis with cesium as cation in solution.[^40^, ^41^, ^42^, ^43^] Furthermore, in studies by Ott *et al*. and Morris *et al*. this ion‐pairing of an ion in solution with a redox‐active species of a MOF was seen to decrease the rate of electron transfer.[^12^, ^13^, ^44^] Moreover, during the CO_2_ reduction reaction with a cobalt porphyrin catalyst immobilized on a pyrolytic graphite electrode it was found that the cation of the electrolyte has an ion‐pairing interaction with the Co‐CO_2_^−^⋅ intermediate that influences the catalysis.^[45]^ Based on these studies the trend observed in this study of ORR activity of Cs^+^>Na^+^≈K^+^≈NMe_4_^+^≈Mg^2+^>Li^+^ with PCN‐224(Co) might indicate that the specific interaction of the cation with the superoxide or peroxide intermediate is influencing catalysis (Figure 10). The trend in ORR catalysis with PCN‐224(Co) with the identity of the cation suggests that the intrinsic rate is most likely limiting the catalytic reaction. Although our data suggests that the activity of the catalytic sites limits the catalytic reaction, we cannot draw any conclusions about the homogeneity of the active sites throughout the samples. Catalysis may be limited to particular pockets – that are sufficiently assessible for electrons and substrate, while other sites within the MOFs may not be active due to the MOF being buried underneath a layer of carbon black, or the active site being locked behind defects in the pore structure. It is important to note that findings presented here are obtained with a particle size of 1–5 μm, and care should be taken in extrapolating to different particle sizes or thin MOF films.

## Conclusions

To investigate the role of the nature and concentration of the electrolyte on the oxygen reduction reaction (ORR) with the PCN‐224(Co) MOF in aqueous electrolyte, a systematic study has been carried out. It was found that the ORR catalysis is slightly influenced by the nature of the ions in solution, providing that the ionic strength is high enough to minimize the resistivity during the measurement. The rate of ORR catalysis with PCN‐224(Co) in an acetate buffer was found to follow the trend Cs^+^>Na^+^≈K^+^≈NMe_4_^+^≈Mg^2+^>Li^+^_._ Moreover, there was no dependency found of the ORR catalysis on the size of the anion, buffer concentration, or oxygen concentration. These findings suggest that ORR catalysis in PCN‐224(Co) is limited by the intrinsic ORR rate at the active site rather than charge transport through the porous structure or oxygen diffusion through the pores. Optimization of ORR catalysis with this MOF should therefore be targeted toward improvement of the electronics at the cobalt active site.

## Experimental details

### Materials and Methods

For synthesis, methyl 4‐formylbenzoate (Sigma Aldrich, 99 %), propionic acid (Alfa Aesar, 99 %), pyrrole (Sigma Aldrich, >98 %), CoCl_2_⋅6H_2_O (Alfa Aesar, 98 %), DMF (VWR), THF (VWR), methanol (VWR), KOH‐pellets (Thermo Fisher, 85 %), HCl (37 %, VWR), ZrCl4 (Alfa Aesar, >99.5 %), and benzoic acid (Alfa Aesar, >99.5 %) were used without further purification. Milli‐Q Ultrapure grade water (>18.2 MΩ cm resistivity) was used for all experiments. ZrCl_4_ was stored in a desiccator. The H_2_O content in DMF was regularly monitored by a Karl‐Fischer titration and was found to be between 1000 and 3300 ppm.

NMR data was recorded on a Bruker Advance 400/101 MHz Ultrashield NMR Spectrometer using residual solvent as an internal standard. Elemental analysis was performed by Mikroanalytisches Laboratorium Kolbe in Oberhausen, Germany. The water content of the DMF was quantified by using a TitroLine 7500 KF trace titrator. Powder X‐ray diffraction (PXRD) spectra were measured on a Rigaku Miniflex II desktop X‐ray diffractometer with Cu Kα radiation (λ=1.5406 Å) with 0.05° steps and a scan speed of 2°/min. Scanning electron microscopy (SEM) images were collected with a Thermo Scientific Apreo scanning electron microscope operating under a high vacuum. Data for images were recorded at an accelerating voltage of 15–20 kV with a 0.1–1.6 nA probe current. SEM samples were prepared by depositing a 5 nm layer of platinum on top of the sample using a Cressington 208 HR sputter coater. ICP‐MS was measured on a NexION® 2000 ICP Mass Spectrometer from Perkin Elmer.

### Synthesis Protocols

#### 5,10,15,20‐(4‐Carboxyphenyl)‐Porphyrin (TCPP)

Methyl‐4‐formylbenzoate (10.9 g; 66.4 mmol) was dissolved in propionic acid (250 ml). While stirring this clear solution, pyrrole (5 ml; 72.1 mmol) was added. The brown solution was refluxed for 1.5 h, after which the mixture was allowed to cool to r.t. The mixture was filtered and washed with acetone (5×15 mL). After drying, the purple solid was dissolved in a solution of 1 : 1 : 1 of THF:methanol:1.9 M KOH in water (225 mL) and this brown solution was refluxed overnight while stirring. The brown solution was acidified by slow addition of HCl (1 M; ~100 mL). The mixture was filtered and washed with ice cold water (3×20 mL). The purple solid was recrystallized by dissolving it in DMF (90 mL), followed by slow addition of water (300 mL). The mixture was filtered and the solid was washed with water (3×20 mL). The product was obtained as a purple solid (2.77 g; 3.5 mmol; 5.3 %). ^1^H NMR (300 MHz, DMSO) δ 8.87 (s, 8H), 8.40–8.32 (m, 16H), −2.94 (s, 2H).

#### Co(5,10,15,20‐(4‐Carboxyphenyl)‐Porphyrin)chloride (CoTCPP)

TCPP (600 mg; 0.76 mmol) and CoCl_2_⋅6H_2_O (2.34 g; 9.8 mmol) were dissolved in DMF (80 mL) and refluxed for 5.5 h. After cooling down to r.t., water (120 mL) was added dropwise. The mixture was filtered and washed with water (3×15 mL). The product was obtained as a brown solid (641 mg; 0.73 mmol; 96 %). Elemental analysis: meas. 64.18 %C, 3.14 %H, 6.19 %N, calc. for [C_48_H_28_ClCoN_4_O_8_+1/2 H_2_O]: 64.62 %C, 3.28 %H, 6.28 %N.

#### PCN‐224(Co)

ZrCl_4_ (37.5 mg, 0.16 mmol), CoTCPP (25 mg; 0.03 mmol) and benzoic acid (1.35 g) were suspended in DMF (4 mL) and sonicated until dissolved (20 min) in a 20 mL screw‐cap scintillation vial (PerkinElmer). The red solution was stirred with a stirrer bar (PTFE, 10×5 mm, VWR) at 400 rpm in a pre‐heated 120 °C oil bath for 24 h. After cooling to r.t., the resulting suspension was poured in a 15 mL polypropylene centrifuge tube (VWR) and was centrifuged at 4000 rpm for 20 min. The supernatant was discarded and the residue was soaked in fresh DMF (14 mL on the tube) twice for 2 h, with the same centrifugation/re‐suspension step in between. The solid was activated by dispersing it in 10 mL DMF with 0.5 mL HCl (8 M in water) in a 20 mL screw‐cap scintillation vial. The mixture was heated overnight in a 100 °C oven. After cooling to r.t., the suspension was poured in a 15 mL polypropylene centrifuge tube and consecutive DMF (2×2 h) and acetone washing cycles (4×24 h) were performed following the same centrifugation/re‐suspension procedure. The final material was dried in a 60 °C vacuum oven overnight to yield a red solid (20 mg).

### Electrochemical Details

#### Electrochemical Set‐Up

All electrochemical measurements were conducted in custom‐made 2‐compartment cells (50 mL) in which the counter electrode is separated from the main compartment *via* a glass frit. Moreover, the reference electrode was separated from the main compartment *via* a Luggin capillary. All glassware used for electrochemical measurements was routinely cleaned from organic materials by overnight soaking in a solution of KMnO_4_ (1 g/L) in H_2_SO_4_ (0.5 M) followed by a 30 min immersion into water containing a few droplets of concentrated H_2_SO_4_ and H_2_O_2_ to remove any manganese traces. Afterward, the glassware was boiled three times for 40 min in water. Milli‐Q Ultrapure grade water (>18.2 MΩ cm resistivity) was used for cleaning of the electrochemical cells. The experiments were performed using a 3‐electrode set‐up equipped with an Autolab PGSTAT 12 Potentiostat with a MSR rotator (Pine instrument) and operated under NOVA software. All electrochemical experiments were conducted under an atmosphere of oxygen (Linde, O_2_ 5.0). Prior to each experiment, the gas was bubbled through the electrolyte solution for at least 15 min. During the measurements, the gas was allowed to continue bubbling in the electrolyte to obtain a stable dissolution of the gas during measurements.

As working electrode, a glassy carbon (GC) disk electrode (d=5 mm) was used in combination with a ChangeDisk Peek electrode holder (E5TQPK, Pine Instrument). The GC electrode was custom‐made by the glassblowers of the Leidse Instrumentenmakers School (LIS) and had a geometric surface area of 0.196 cm^2^. The GC electrode was polished on a Struers LaboPol‐30 polishing machine by rotating the polishing cloth (Dur‐type) at 200 rpm clockwise and moving the electrode in a counterclockwise circular motion over the pad. The GC electrode was first polished with a diamond suspension (1.0 μm, DiaPro, Struers) on the polishing cloth for 1 min, after which the electrode was rinsed with isopropanol and water. Next, the electrode was polished on a different polishing cloth with a silica suspension (OPS Non‐dry, Struers) for 1 min, after which the electrode was rinsed with water and sonicated in water for 10 minutes. As counter electrode, a coiled Au wire (MaTeck, 0.6 mm) was used. This Au coil was flamed and rinsed with water prior to each measurement. As reference electrode either a Pt gauze was used while bubbling H_2_ (Linde, H_2_ 5.0) through the buffer solution in the Luggin capillary, or an Ag/AgCl electrode (3 M KCl, E^0^=0.197 V vs. NHE, Metrohm) was used.

#### Electrolytes

CH3COOH (Suprapur, Supelco), NaCH3COO (TraceSELECT, ≥99.999 %), LiCH3COO⋅H_2_O (Puratronic, 99.998 %), KCH3COO⋅H_2_O (Puratronic, 99.997 %), CsCH3COO (Thermo Scientific Chemicals, 99.998 %), Mg(CH3COO)_2_⋅4H_2_O (Puratronic, 99.997 %), NMe_4_OH (Thermo Scientific Chemicals, 25 % w/w, 99.9999 %,), NaOH⋅H_2_O (Suprapur, 99.9 %), NaNO_3_ (Suprapur, 99.99 %), NaClO_4_⋅H_2_O (Emsure), and Na_2_SO_4_ (Suprapur, 99.99 %) were used without further purification. All electrolyte salts are stored in a desiccator under vacuum to prevent hydration. The pH of the buffer solutions was measured with a HI 4222 pH meter (Hanna Instruments), calibrated with IUPAC standard buffers (Radiometer analytical).

#### Catalyst Ink Preparation

For the catalyst ink preparation, Nafion (Merck, 5 wt% in alcohols), carbon black (CB, Alpha Aesar, acetylene 50 % compressed), and acetone (Merck) were used. The ink was made by combining PCN‐224(Co) (1.5 mg), CB (1.5 mg), Nafion (20 μL) and acetone (180 μL) in a 1.5 mL autosampler vial. The ink mixture was sonicated for 20 min to allow mixing. Prior to dropcasting the ink on the electrode, the mixture was placed on a vortex for 10 seconds to allow for maximum homogeneity in the suspension. Before the ink was dropcasted on the electrode, the cleanliness of the GC electrode was verified by measuring a rotating disk electrode cyclic voltammogram in a 0.2 M acetate buffer. Once it was established that the carbon electrode behaved as expected, the electrode was rinsed with water and dried with a precision wipe (Kimtech Science), after which 10 μL of the ink was dropcasted on the electrode. The dropcast was allowed to dry for at least 15 min in air, after which the measurements were carried out.

#### Rotating Disk Electrode Cyclic Voltammetry

Rotating disk electrode cyclic voltammetry (RDE CV) measurements were conducted with a scan rate of 50 mV/s, step size of 5 mV, and rotation rate of 1600 rpm. Due to the hydrophobicity of the CB in the ink solution, a small air bubble often remained on the electrode after submersion of the electrode in the buffer. A single RDE CV measurement of three cycles was carried out in which the bubbles were removed. Next, an RDE CV measurement was conducted of which the second forward scan was used for calculations presented in this work.

## Conflict of Interests

The authors declare no conflict of interest.

1

## Supporting information

As a service to our authors and readers, this journal provides supporting information supplied by the authors. Such materials are peer reviewed and may be re‐organized for online delivery, but are not copy‐edited or typeset. Technical support issues arising from supporting information (other than missing files) should be addressed to the authors.

Supporting Information

## Data Availability

The data that support the findings of this study are available in the supplementary material of this article.
