# Supplementary material for: Effect of the Electrolyte on the Oxygen Reduction Reaction with a MOF Embedded Co‐Porphyrin
Source: ChemSusChem. 2025 Jan 30;18(10):e202402295. doi: 10.1002/cssc.202402295 (PMC12094139; doi:10.1002/cssc.202402295)
Supplement: Supplementary file 1 — Supporting Information [file CSSC-18-e202402295-s001.pdf]

ChemSusChem

Supporting Information

Effect of the Electrolyte on the Oxygen Reduction Reaction with a MOF Embedded Co-Porphyrin

Dana Rademaker, Stefania Tanase, and Dennis G. H. Hetterscheid*

Effect of the Electrolyte on the Oxygen Reduction Reaction with a MOF embedded Co-porphyrin

Dana Rademaker, Stefania Tanase, and Dennis G.H. Hetterscheid

Supporting information

Contents

2. Calculate turn-over frequency 2
3. Buffer dependence study with CoTCPP 2
3. LSV traces of PCN-224(Co) in various cation acetate buffers 4
4. LSV traces of PCN-224(Co) in electrolytes with various anions 5
5. Rotation rate dependence PCN-224(Co) 6
6. References 6

2. Calculate turn-over frequency

The turn-over frequency (TOF) was calculated for the two-electron reduction of oxygen to hydrogen peroxide. In Chapter 3 the selectivity of the ORR with PCN-224(Co) towards H_2O_2 was confirmed. Therefore, the equation to derive the TOF is:[1]

$$TOF = \frac{moles\ of\ H_2O_2\ formed}{moles\ of\ cobalt\ sites \times time} = \frac{i_{cat}/(2F)}{N}$$

With i_{cat} as the catalytic current (A or C/s), F as Faradays constant (96485 C mol^{-1}), and N as total moles of cobalt sites.

The loading on the electrode is 75 µg of PCN-224(Co), which equals 0.055 µmol of cobalt sites. The TOF is determined as a function of all cobalt sites.

3. Buffer dependence study with CoTCPP

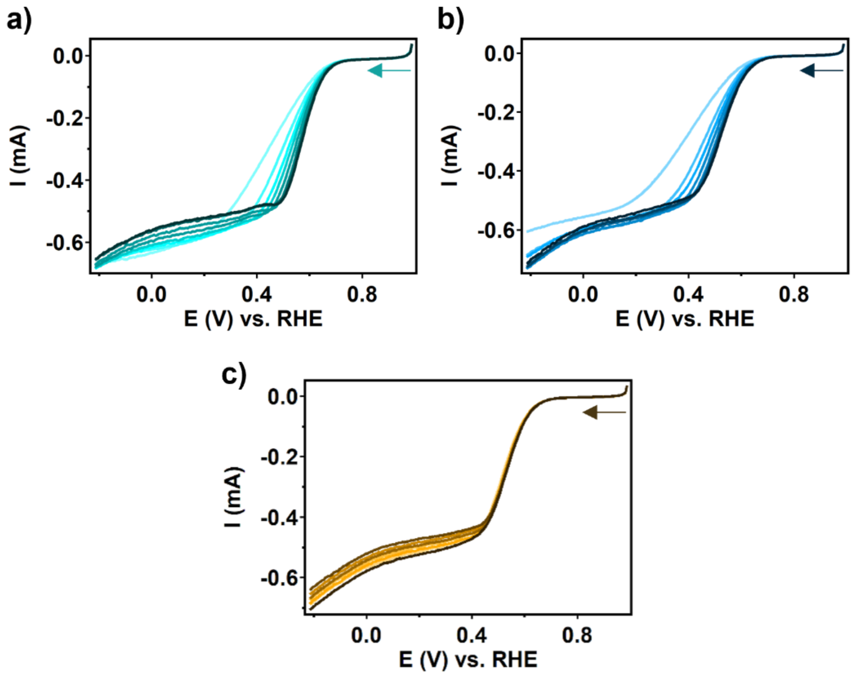

Figure S1. RDE LSV traces of CoTCPP in (a) sodium acetate buffer (pH 4.7) with concentrations of 50, 100, 150, 200, 300, 400, 500, and 600 mM (from light to dark); (b) sodium acetate buffer (pH 4.7, 50 mM) with increasing $NaNO_3$ concentrations of 0, 25, 50, 75, 125, 175, 225, and 275 mM (from light to dark); and (c) in an electrolyte of $NaNO_3$ and sodium acetate buffer at a constant ionic strength of 300 mM, while changing the sodium acetate buffer to 50, 100, 150, 200, 300, 400, and 500 mM (from light to dark). Measured with 50 mV/s scan rate and 1600 rpm rotation rate under an oxygen atmosphere.

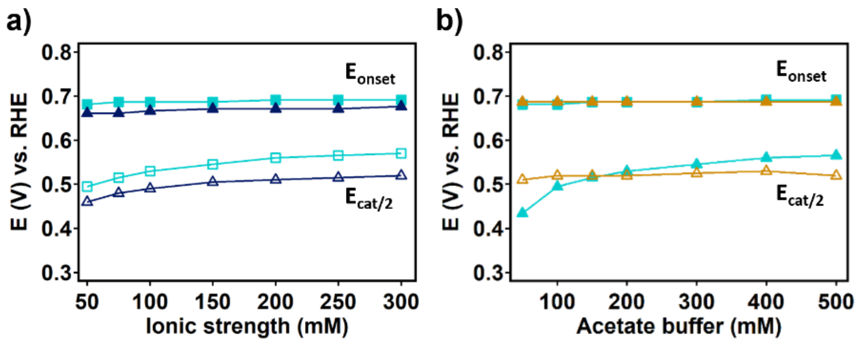

Figure S2. Analysis of RDE LSV traces of CoTCPP with (a) E_{onset} and $E_{cat/2}$ as a function of the ionic strength of the sodium acetate dependence between 50 – 300 mM ionic strength of sodium acetate buffer (teal) and the ionic strength dependence with 50 mM of sodium acetate and $NaNO_3$ as supporting electrolyte (dark blue). (b) E_{onset} and $E_{cat/2}$ as a function of the sodium acetate buffer concentration

between 50 – 300 mM ionic strength of sodium acetate buffer (teal) and the sodium acetate buffer dependence with supporting $NaNO_3$ electrolyte to achieve a fixed ionic strength of 300 mM (orange).

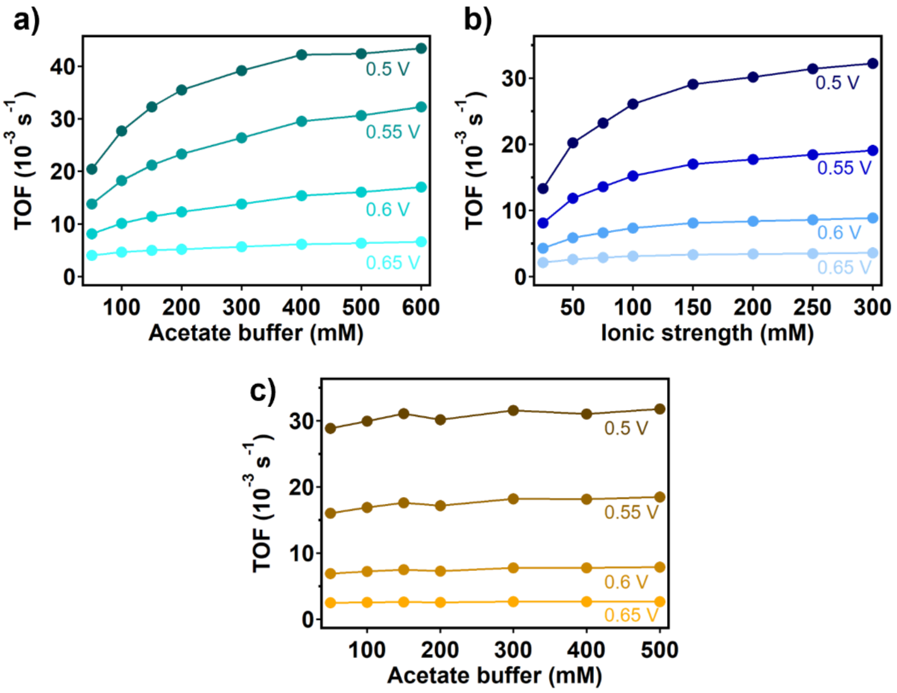

Figure S3. The TOF at 0.35, 0.4, 0.45, and 0.5 V vs. RHE (a) as a function of the sodium acetate buffer concentration between 50 – 300 mM ionic strength of sodium acetate buffer, (b) as a function of the ionic strength with a constant 50 mM of sodium acetate and variable $NaNO_3$ concentration as supporting electrolyte, and (c) as a function of the sodium acetate buffer concentration of the sodium acetate buffer dependence with supporting $NaNO_3$ electrolyte to achieve a fixed ionic strength of 300 mM.

3. LSV traces of PCN-224(Co) in various cation acetate buffers

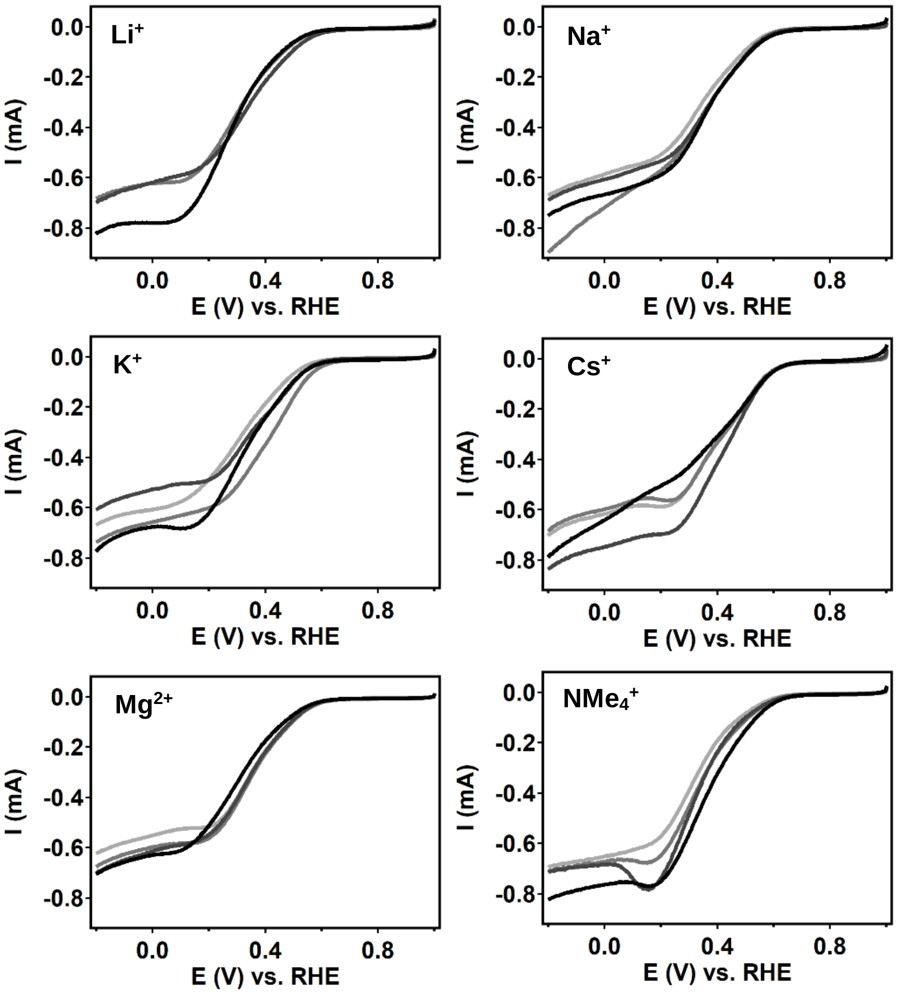

Figure S4. RDE LSV traces of PCN-224(Co) in 500 mM acetate buffer with lithium, sodium, potassium, cesium, magnesium, or tetramethylamine as cationic species. Measured with 50 mV/s scan rate and 1600 rpm rotation rate under an oxygen atmosphere.

Table S1. Turn-over frequencies of ORR with PCN-224(Co) in different cation acetate buffer solutions as a function of the applied potential.[2]

**Metal Cation Properties**			**TOFs (10^{-3} s^{-1}) in case of [Cation]OAc.**			
Ion	Ionic Radius (Å)	Hydrated Radius (Å)	0.5 V	0.45 V	0.4 V	0.35 V
Li^+	0.94	3.82	7.0 ± 1.7	11.8 ± 2.4	17.9 ± 2.5	25.4 ± 2.6
Na^+	1.17	3.58	10.7 ± 1.1	17.0 ± 1.5	23.9 ± 2.0	32.2 ± 2.8
K^+	1.49	3.31	10.3 ± 3.7	17.1 ± 5.4	24.2 ± 6.5	31.5 ± 7.1
Cs^+	1.86	3.29	17.4 ± 1.3	25.6 ± 2.7	33.0 ± 4.5	40.9 ± 6.1
Mg^{2+}	0.72	4.28	8.3 ± 1.0	13.6 ± 1.6	20.0 ± 2.2	28.2 ± 2.9
NMe_4^+	2.85	3.47	10.6 ± 2.7	16.3 ± 4.1	23.7 ± 5.2	33.5 ± 6.1

4. LSV traces of PCN-224(Co) in electrolytes with various anions

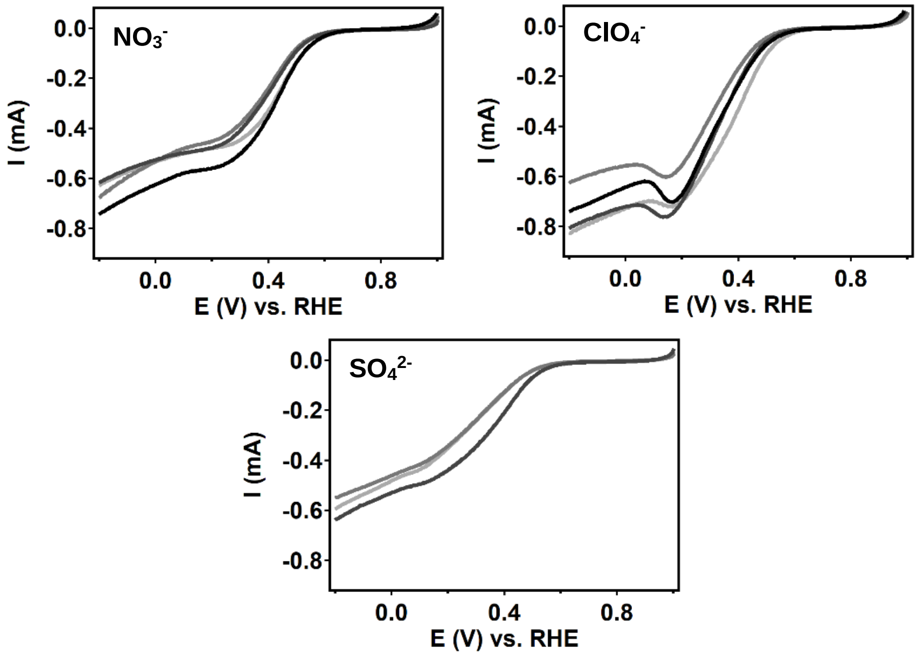

Figure S5. RDE LSV traces of PCN-224(Co) in 50 mM sodium acetate buffer with addition of sodium nitrate, sodium perchlorate, or sodium sulphate to obtain a 250 mM ionic strength solution. Measured with 50 mV/s scan rate and 1600 rpm rotation rate under an oxygen atmosphere.

Table S2. Turn-over frequencies of ORR with PCN-224(Co) in different sodium anion solutions as a function of the applied potential.[2]

**Anion Properties**			**TOFs (10^{-3} s^{-1}) in case of Na[Anion].**			
Ion	Ionic Radius (Å)	Hydrated Radius (Å)	0.5 V	0.45 V	0.4 V	0.35 V
CH_3COO^-	1.62	2.17	10.7 ± 1.1	17.0 ± 1.5	23.9 ± 2.0	32.2 ± 2.8
NO_3^-	1.79	2.23	10.7 ± 2.7	19.0 ± 4.2	28.2 ± 5.2	35.7 ± 5.3
ClO_4^-	2.50	2.69	7.1 ± 2.7	13.8 ± 4.7	23.1 ± 6.5	33.2 ± 7.1
SO_4^{2-}	2.30	2.73	4.8 ± 1.6	9.2 ± 3.0	14.9 ± 4.5	20.7 ± 5.4

5. Rotation rate dependence PCN-224(Co)

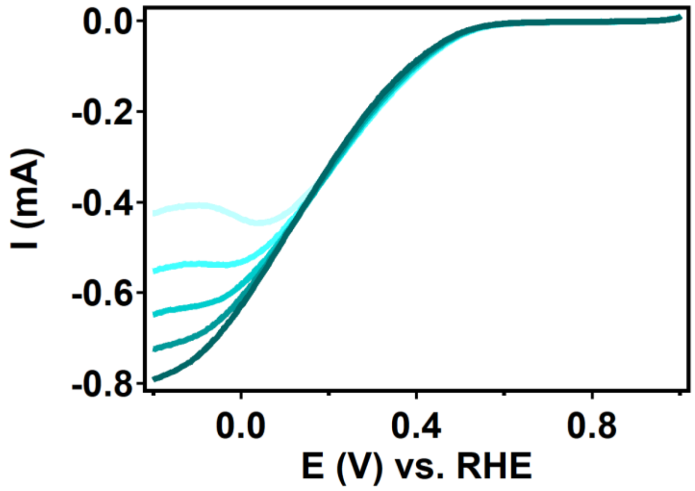

Figure S6. RDE LSVs at rotation rates between 400 and 2000 rpm with 400 rpm increments from light to dark colour with PCN-224(Co). Measured in 0.2 M acetate buffer (pH 4.7) with 50 mV/s scan rate under an oxygen atmosphere.

6. References

[1] K. Dong, J. Liang, Y. C. Ren, Y. Y. Wang, Z. Q. Xu, L. C. Yue, T. S. Li, Q. Liu, Y. L. Luo, Y. Liu, S. Y. Gao, M. S. Hamdy, Q. Li, D. W. Ma, X. P. Sun, *J. Mater. Chem. A* **2021**, *9*, 26019-26027.

[2] a) A. G. Volkov, S. Paula, D. W. Deamer, *Bioelectrochem. Bioenerg.* **1997**, *42*, 153-160; b) Y. Marcus, *J. Chem. Soc., Faraday trans.* **1991**, *87*, 2995-2999.
